# Microbial metagenomics in the Baltic Sea: Recent advancements and prospects for environmental monitoring

**DOI:** 10.1007/s13280-015-0663-7

**Published:** 2015-05-28

**Authors:** Karolina Ininbergs, Birgitta Bergman, John Larsson, Martin Ekman

**Affiliations:** Science for Life Laboratory, Department of Ecology, Environment and Plant Sciences, Stockholm University, Box 1031, 171 21 Solna, Sweden; Science for Life Laboratory, Linnaeus University Kalmar, 171 21 Solna, Sweden

**Keywords:** Microbial metagenomics, Baltic Sea, Environmental monitoring, Brackish microbial communities, Bacterial diversity

## Abstract

Metagenomics refers to the analysis of DNA from a whole community. Metagenomic sequencing of environmental DNA has greatly improved our knowledge of the identity and function of microorganisms in aquatic, terrestrial, and human biomes. Although open oceans have been the primary focus of studies on aquatic microbes, coastal and brackish ecosystems are now being surveyed. Here, we review so far published studies on microbes in the Baltic Sea, one of the world’s largest brackish water bodies, using high throughput sequencing of environmental DNA and RNA. Collectively the data illustrate that Baltic Sea microbes are unique and highly diverse, and well adapted to this brackish-water ecosystem, findings that represent a novel base-line knowledge necessary for monitoring purposes and a sustainable management. More specifically, the data relate to environmental drivers for microbial community composition and function, assessments of the microbial biodiversity, adaptations and role of microbes in the nitrogen cycle, and microbial genome assembly from metagenomic sequences. With these discoveries as background, prospects of using metagenomics for Baltic Sea environmental monitoring are discussed.

## Introduction

Abundant and ubiquitous environmental microorganisms are important drivers of global biogeochemical cycles, and understanding factors controlling their abundance, activities, and diversity is therefore a major area of research. Studying microbes in situ, however, is challenging. Microbial communities are composed of mixed and diverse assemblages, often reside in hard to sample habitats, and a great majority is un-cultivable in the laboratory. The development of high throughput sequencing (HTS) technologies during the last decade has therefore in many ways revolutionized the study of natural communities of microbes, now characterized as the “unseen majority.” This technology allows, in a cost-effective way, the analysis of diversity, metabolic functions, and biological interactions in complex, uncultured microbial communities. Extraction of DNA from mixed communities of microbes, followed by HTS, has greatly increased our understanding of the fundamental roles played by terrestrial, aquatic, and human-associated microbiota (Fierer et al. 2007; Zinger et al. 2011; Huttenhower et al. 2012). Pioneering work analyzing sequenced environmental DNA (eDNA) from marine bacterial communities led to the discovery that much of the microbial diversity in the world’s oceans, from surface to deep waters, had been severely underestimated (Venter et al. 2004; DeLong et al. 2006). Important findings from HTS of eDNA or RNA include the discovery of novel genes, proteins, and microbial species (Yooseph et al. 2007; Gilbert et al. 2008), and findings related to the role of microbes in global biogeochemical cycles of carbon and nitrogen (Frias-Lopez et al. 2008). More recent analyses have also shown that genomic plasticity and metabolic versatility of microorganisms are the basis for bacterial adaptation in marine ecosystems (Konstantinidis et al. 2009; Lauro et al. 2009; Yooseph et al. 2010). Hence, the development and introduction of these potent technologies have enabled a much more detailed view of microbial communities and their functions in natural settings and have profoundly changed our perception of microbial life, genome evolution, and minimal requirements for life (Karl 2007; Giovannoni et al. 2014).

HTS technology-based analyses of natural microbial communities can be divided into two sub-classes. The more commonly used “HTS-signature-gene” approach surveys eDNA for single marker gene distributions and abundances and often uses the conserved 16S rRNA gene, encoding the small subunit of the prokaryotic ribosome. In contrast, the metagenomic shotgun approach potentially exposes the majority of genes/genomes present in eDNA extracted from natural microbial populations. Due to the non-targeted nature of the metagenomic approach, substantial amounts of diverse genetic data and information on functional potential of entire microbial communities may be obtained. Metagenomics, however, holds its own biases. As a result of differences in genome sizes and genetic sequence (e.g., high or low GC content), DNA from different genes and organisms are not uniformly covered during sequencing, nor can all sequences be correctly annotated (identified) due to lack of experimental evidence for protein-coding sequences and the still limited number of sequenced microbial genomes available in databases. Metagenomics is increasingly often combined with metatranscriptomics for which all RNA molecules in a natural sample are targeted, expanding the scope of metagenomics by also providing information on gene expression.

Most early metagenomic studies focused on oceans, while smaller seas, freshwater systems, and, in particular, brackish-water transitions until recently have remained poorly investigated using HTS. The Baltic Sea is the world’s second largest body of brackish water and represents one of the most intensely researched and monitored aquatic environments, with a time series of hydrographic data measured routinely for over 100 years (Fonselius and Valderrama 2003). The contemporary Baltic Sea is, since the turn of the twentieth century, negatively affected by anthropogenic disturbances, specifically urban and agricultural derived eutrophication, fueling phytoplankton blooms causing increased anoxia and hypoxia in deep waters (Savage et al. 2010; Carstensen et al. 2014). In addition, the Baltic Sea offers steep gradients in salinity and key-nutrient concentrations. These gradients are semi-constant over time but change dramatically over a short geographical distance, giving rise to a challenging environment for many marine and freshwater organisms. Paired with geographic isolation, this has resulted in low species and genetic diversity among metazoans (e.g., fish, seals) and macrophytes (e.g., macro-algae) (Johannesson and Andre 2006). The diversity and biogeography of microbes in the Baltic Sea and associated waters have, however, attracted considerably less research.

Here, we provide an updated account on how the introduction of HTS-based analyses into Baltic Sea microbial research has contributed to the understanding of Baltic Sea microbes. Recent data collectively illustrate that Baltic Sea microbes are both unique and highly diverse, and well adapted to this brackish water ecosystem. To widen the scope, the Baltic Sea findings are placed in the context of metagenomic microbial findings in oceans in general. Finally, the ecological significance of microbes in any environment suggests a need for implementation of HTS data into future environmental monitoring programs, prospects of which are discussed here in a Baltic Sea perspective.

## Environmental sequencing of Baltic Sea microbes

Earlier reports on Baltic Sea microorganisms focused largely on quantifying abundance and activity in relation to physicochemical parameters (see e.g., Hagström and Larsson 1984; Gast and Gocke 1988; Rheinheimer et al. 1989). The introduction of molecular-based approaches expanded our knowledge of microbial community composition, seasonal succession, and phylogenetic diversity, revealing for instance temporal patterns for specific bacterial phylotypes (Pinhassi et al. 1997; Pinhassi and Hagström 2000) and strong influence of freshwater phyla on brackish water communities (Riemann et al. 2008). A pre-HTS-era metagenomic analysis (using a cloning approach) of Baltic Sea sediment microbial communities was published in 2007 (Hårdeman and Sjöling 2007), while the first HTS-signature-gene-based (pyro-sequencing) study appeared in 2010 (Andersson et al. 2010). This study targeted bacterioplankton at the Landsort Deep, the deepest (459 m) location, and long-term monitoring site in the Baltic Sea (Baltic proper). Subsequently, a number of HTS-based studies of the Baltic Sea microbial life have followed. As seen in Table 1, six studies are based on the HTS-signature-gene approach and four on random metagenomic sequencing of all genes. The work of Feike et al. (2012) stands out by being the only purely metatranscriptomic analysis, and yet another recent study describes genome assembly based on metagenomic sequences (Herlemann et al. 2013).

**Table 1:** Summary of HTS studies of microorganisms in the Baltic Sea

No. of sampling locations/time points	Sequencing platform	Target DNA/RNA—reads/genes	Number of sequenced reads	Reference
21/1	454 Pyro-sequencing	DNA all genes and—16S rRNA	~20 900 000 1 247 371 16S rRNA	Dupont et al. (2014)
21/1	454 Pyro-sequencing & Illumina	DNA—all cyanobacterial genes	698 865 (454 reads) 7 458 747 084 (Illumina reads)	Larsson et al. (2014)
60/Mesocosm	454 Pyro-sequencing	DNA—16S rRNA	135 037	Herlemann et al. (2014)
3/1	454 Pyro-sequencing	DNA—all genes	1 205 630	Thureborn et al. (2013)
16/Mesocosm	454 Pyro-sequencing	DNA—16S rRNA	97 582	Dinasquet et al. (2013)
215/1	454 Pyro-sequencing	DNA—all genes (genome binning)	37 658 923	Herlemann et al. (2013)
10/12	454 Pyro-sequencing	DNA/RNA—*nifH*	79 090	Farnelid et al. (2013)
5 Sampling procedures/1	454 Pyro-sequencing	RNA—all genes	190 262	Feike et al. (2012)
213/1	454 Pyro-sequencing	DNA—16S rRNA	224 076^a^	Herlemann et al. (2011)
11/1	454 Pyro-sequencing	DNA—16S rRNA	36 108	Koskinen et al. (2011)
1/8	454 Pyro-sequencing	DNA—16S rRNA	162 256	Andersson et al. (2010)

^a^Calculated based on sample average

## Bacterial biogeography and diversity

In addition to steep horizontal and vertical concentration gradients related to salinity and key-nutrients, such as nitrogen and phosphorous, there is also a pronounced seasonal variation in both nutrient concentrations and temperature in the Baltic Sea. The effects of these variables were investigated by Andersson et al. (2010) using a 16S rRNA HTS approach to study bacterioplankton communities at Landsort Deep. A pronounced influence of both phosphorous and temperature on the microbial community, notably composed of characteristic freshwater bacteria such as actinobacteria, betaproteobacteria, and verrucomicrobia, was found. While some early studies suggested that salinity may influence the Baltic Sea bacterial growth and biogeography (Väätänen 1980; Heinanen 1991), the extent of this effect was not realized until the first large-scale 16S rRNA gene inventory along the entire Baltic Sea salinity gradient was performed (Herlemann et al. 2011). A subsequent comprehensive metagenomic survey substantiated the strong structuring of the bacterial community composition along the Baltic salinity gradient and also included the freshwater Lake Torne Träsk and the marine waters off the Swedish west coast as additional reference points (Dupont et al. 2014). Among eubacteria, a clear dominance of actinobacteria was apparent in the low-salinity Bothnian Bay in the northern Baltic Sea, while a shift toward dominance of proteobacteria (mainly alpha and gamma) was apparent at higher salinities (including at the Swedish west coast). As in most global oceans, the dominant bacteria in the Baltic Sea were alphaproteobacteria of the SAR11 clade (Morris et al. 2002; Dupont et al. 2014). Overall the community compositions at the phylum level in the two studies were largely in agreement and both reported a unique autochthonous brackish bacterial population present at intermediate salinity stations, including strains of SAR11 and picocyanobacteria (Herlemann et al. 2011; Dupont et al. 2014).

The variation in bacterial community composition seen in the Baltic Sea along the salinity transect is considerably more dramatic than in most oceanic habitats (Dupont et al. 2014). A comparative network-analysis of metagenomes (both with respect to taxonomy and functional potential) collected at various depths (from surface to anoxic sediments) in the Baltic Sea and 27 metagenomes from 11 sites worldwide showed that the Baltic Sea bacterial communities clustered primarily with metagenomes from the western English Channel (Thureborn et al. 2013). Unique to the Baltic Sea, however, was the community derived from the metagenome collected at the oxic-anoxic interface, being an outlier with few taxonomic similarities to any other community (Thureborn et al. 2013). It should, however, be emphasized that the reference metagenomes used in these analyses were all from marine environments (except two that were from terrestrial), and that only one geographic site in the Baltic Sea was included. The increased availability of metagenomes from the whole Baltic Sea salinity transect now warrants expanded comparative studies.

Other findings from comparing HTS analyses from the Baltic Sea to other marine environments relate to the diversity of the Baltic Sea microbial community. In one of the first HTS/16S rRNA based studies, a lower bacterial diversity was observed in the central eastern Baltic Sea (northern Baltic proper/Gulf of Finland) compared to that of some investigated fully marine oceanic habitats (Koskinen et al. 2011). However, later HTS-based analyses (16S rRNA and metagenomic) of the genetic diversity of bacteria along the whole longitudinal expansion of the Baltic Sea did not show any such reduced diversity at intermediate salinities (Herlemann et al. 2011; Dupont et al. 2014). In contrast, these studies rather demonstrate a surprisingly high microbial diversity throughout the Baltic Sea. This suggests that Baltic Sea microorganisms are less impaired by genetic isolation than are macro-organisms (Johannesson and Andre 2006), presumably underpinned by a combination of short microbial doubling times (hours/days) and comparatively small and flexible genomes. A characteristic of microbes is also frequent horizontal gene transfer events between sympatric microbes, a phenomenon that was recently investigated in Baltic Sea picocyanobacteria (Larsson et al. 2011).

## Microbial community function: From gene frequencies to genome assembly

Metagenomic data may be used to predict metabolic potentials of microbes as they expose existing gene repertoires and related metabolic processes. Although other factors such as regulation of gene transcription and enzyme activities, and the availability of substrates, are of critical importance, the relative frequencies of specific genes may point to their functional importance in an environment. While the effect of salinity in shaping microbial communities is well known (see e.g., Lozupone and Knight 2007; Campbell and Kirchman 2013), the mechanism behind salinity being such a strong barrier to cross for bacteria is not. However, a recent metagenomic analysis, encompassing the Baltic Sea salinity gradient, revealed that salinity does not only influence the distribution of traits such as ion transporters (e.g., Na, K) and biosynthesis and transport of compatible solutes, but also bacterial metabolic core functions such as respiration, glycolysis, and cofactor biosynthesis (Dupont et al. 2014). Notably, analogous metabolic pathways, with approximately the same outcome but via different intermediate metabolites and genes, were found to have opposite abundance patterns along the salinity gradient. For instance, the glycolytic Entner Doudoroff (ED) pathway dominated at high salinity while the Embden–Meyerhof (EM) pathway at low salinity (Dupont et al. 2014). Low-salinity adapted bacteria overall use pathways with a higher ATP yield than bacteria in marine environments. These metabolic differences may explain the distinct divide known to exist between fresh and marine microbial communities. It further suggests that adaptation to a lower salinity may be based on a core gene set with higher energy yield. This discovery now calls for further exploration.

Other important findings from metagenomic functional analyses of the Baltic Sea microbes relate to the impact of eutrophication and pollution. Nutrients (nitrogen and phosphorus) are the second most important factor (besides salinity) in shaping the distribution of microbial taxa and their functional potential in Baltic Sea surface water communities (Dupont et al. 2014). More specific information on the influence of eutrophication and pollution was obtained from a metagenomic study of the Landsort Deep microbial community (Thureborn et al. 2013). The functional gene repertoire showed a comparatively high abundance of microbial genes involved in attachment to and degradation of organic carbon and in heavy metal resistance (e.g., against cobalt, cadmium, and zinc). These findings are likely related to organic matter deposition and the high concentrations of metals in sediments at this site (Thureborn et al. 2013). Overall, the resulting gene diversity at this specific site appears to be shaped by anthropogenic pollution and eutrophication. However, it should be noted that the deeper waters at this site offer radically different conditions (hypoxia or anoxia) compared to the rest of the Baltic Sea, affecting the overall bacterial biodiversity dramatically (Dupont et al. 2014). The effect of dissolved organic carbon (DOC) on the Baltic Sea bacterial community was also recently investigated by combining mesocosms and metagenomic analyses (Dinasquet et al. 2013; Herlemann et al. 2014). While a weak effect of the DOC on structuring the community was the norm, some taxa were clearly influenced. This approach may be an efficient tool to evaluate details in successional changes as a response to nutrient regimes.

To further explore putative functions, (meta-)genomic assembly data in the form of contigs, i.e., longer genomic sequences obtained by aligning partly overlapping sequence reads, may be analyzed. For example, a recent analysis of light-harvesting genes in Baltic Sea identified a novel gene cluster in the picocyanobacterial population (Larsson et al. 2014). These cyanobacteria, with a cell size <2 µm, are major primary producers in oceans (Scanlan et al. 2009). This is also the case in the Baltic Sea where picocyanobacteria may constitute up to 80 % of the cyanobacterial population (Stal et al. 2003; Hajdu et al. 2007). Metagenomic analyses show that the Baltic Sea picocyanobacteria are dominated by strains belonging to the genera *Synechococcus* and *Cyanobium* (unpublished results) and that members of the dominant *Synechococcus* clade harbor a novel gene cluster encoding proteins for a unique set of light-harvesting antennae, i.e., pigment-associated phycobilisomes, not previously found in cyanobacteria (Larsson et al. 2014). The organization of the gene cluster suggests the involvement of multiple horizontal gene transfer events. The Baltic Sea picocyanobacteria may have evolved a set of phycobiliproteins with a potentially unique absorption spectrum, to specifically match light conditions offered by the Baltic Sea. These findings exemplify how “meta-omic” datasets can provide novel insights and generate hypotheses-driven research, particularly targeting processes in the large segment of still non-cultivable aquatic microbes, including those in the Baltic Sea.

With recent developments in bioinformatic analysis of metagenomic data, it is possible to assemble not only contigs of a limited length but also assemble near complete genomes of abundant organisms (Iverson et al. 2012). For example, metagenomic time-series samples from the Baltic Sea were used to assemble a genome from an aquatic phylotype of the verrucomicrobia *Spartobacteria* (Herlemann et al. 2013), which is one of the dominant organisms in the Baltic Sea bacterial community during the summer (Herlemann et al. 2011). Analysis of the assembled *Spartobacteria* genome gave important information about the metabolic capacity of the bacterium, including the presence of 23 glycoside hydrolases, giving the bacterium the capacity to metabolize a number of different carbohydrates and suggesting a potentially important role in carbon cycling. Based on patters of co-occurring abundances, it was further suggested that the carbon was mainly derived from cyanobacterial blooms. It is clear that this approach can take the metagenomic scope even further and provides an important step in increasing the number of microbial genomes available, one of the prerequisites for correct annotation of metagenomic sequences.

## The nitrogen cycle: From anoxic zones to surface waters

Today, the Baltic Sea suffers from large and persistent anoxic bottom zones. This is partly a natural phenomenon caused by strong stratification, which prevents vertical mixing. Eutrophication of the Baltic Sea has increased the area of these zones (Carstensen et al. 2014), and they today constitute the largest anthropogenically induced hypoxic area in the world.

The nitrogen cycle consists of microbially mediated transformations of nitrogen, some of which are dependent on reducing conditions (e.g., denitrification and anamox). Research concerning hypoxic environments in the Baltic Sea has therefore often targeted these processes, more recently using HTS technologies. Microbial metagenomes from the Landsort Deep water column illustrate a stratification of the microbial functional capacities along the depth and oxygen profile (Thureborn et al. 2013; Dupont et al. 2014). While genes for the anamox reaction were absent, high frequency of genes involved in denitrification prevailed at the deepest anoxic sites (Thureborn et al. 2013). It was furthermore suggested that the denitrification at this depth was primarily carried out by chemolitotrophic (sulfur oxidizing) denitrifying epsilonbacteria. Later, also Dupont et al. (2014) observed a high prevalence of epsilonbacteria in these specific waters. Together the findings suggest an important role of these organisms in denitrification at the Landsort Deep.

The substrate for epsilonbacterial denitrification (nitrate) was in the Thureborn et al. (2013) suggested to originate from aerobic ammonia oxidizing thaumarchaeota. High abundance of ammonia oxidizing thaumarchaeota was indeed previously observed in the suboxic zones (70–120 m depth) of the central Baltic Sea (Labrenz et al. 2010). More recently, metatranscriptome analyses substantiated these findings by showing that the transcript level of ammonia oxidation genes (*amo*A, *amo*B, and *amo*C) was high in the suboxic zones of both Landsort and Gotland Deep (Feike et al. 2012). The nitrifying potential of pelagic archaea was first demonstrated by an early metagenomic study (Venter et al. 2004). Since then ammonia-oxidizing archaea have been found to be widely distributed in the world’s oceans and likely play a significant role in the global nitrogen cycle (Erguder et al. 2009).

Fixation of atmospheric dinitrogen is a microbial process that has received considerable research attention (Gruber 2005). This is particularly the case for the Baltic Sea, with its typical massive summer blooms of nitrogen-fixing cyanobacteria (Stal et al. 2003, Kahru and Elmgren 2014). In fact, the nitrogen fixation by the large filamentous cyanobacterial blooms represents the second largest source of “new” nitrogen (N) input into the Baltic Sea after riverine load (Larsson et al. 2001). The principal enzyme that catalyzes nitrogen fixation, nitrogenase, is encoded by highly conserved *nif* genes (*nifKDH* encoding the structural protein), and these genes are obvious targets in metagenomic and metatranscriptomic surveys. Metagenomic surveys for *nif* genes in the surface waters of the ocean have found surprisingly few sequence reads, despite high rates of nitrogen fixation repeatedly recorded by bloom-forming cyanobacteria (Johnston et al. 2005). The reasons for rare *nif* gene findings in metagenomes may be due to a comparatively restricted distribution of these genes among organisms in the massive microbial metagenomic datasets (Johnston et al. 2005). To address this difficulty, it has been suggested that to properly expose all potential nitrogen-fixers, a minimum set of six *nif* genes should be targeted, namely *nifHDK* and *nifENB*, (Dos Santos et al. 2012). Even when including these *nif* genes in metagenomic analysis of Baltic Sea surface waters, few *nif* sequences were retrieved (Thureborn et al. 2013). This was explained by pre-bloom sampling and to the use of a pre-filtration step (<3.0 µm), which may have excluded the dominant larger nitrogen-fixing filamentous cyanobacteria. A considerably higher number of *nif* gene sequences were later retrieved from a Baltic Sea metagenomic dataset sampled in July and including larger sized microbes (3.0–200 µm) (Dupont et al. 2014).

Despite the relatively few *nif* sequences retrieved from surface waters in the Baltic Sea, *nif* gene abundances increased with depth at Landsort Deep (Thureborn et al. 2013). This suggests the involvement of heterotrophic organisms, in this case, sulfate-reducing Deltaproteobacteria (comprising 36 % of the *nif* genes at this site), in Baltic Sea nitrogen fixation. Combining *nifH* HTS analyses, gene expression measurements and nitrogen fixation rate determinations for Baltic Sea microbes (Farnelid et al. 2013) showed that heterotrophic nitrogen fixation may account for up to 6 % of the total annual nitrogen fixation. Heterotrophic nitrogen fixation rates have recently been documented in hypoxic waters of, for example, the eastern tropical South Pacific (Fernandez et al. 2011) and the Southern Californian Bight (Hamersley et al. 2011). Hence, heterotrophic nitrogen fixation may constitute an overlooked component of the nitrogen cycle not only in the Baltic Sea but also in other oceans. Additional spatial and temporal studies are now warranted to deepen our knowledge on nitrogen fixation and, in particular, on the variety of microbial *nif* gene operators in Baltic Sea waters, besides the well-known photoautotrophic cyanobacteria assumed to dominate.

## Metagenomics in Baltic Sea monitoring

HTS-based methods have huge capacity to provide detailed and all-encompassing information on microbial identity and potential function. In turn, this creates great potential for sequencing-based monitoring programs. Major advantages include the improved accessibility (i) to a widened coverage of uncultured organisms, (ii) to functional genes targeting specific metabolic processes of relevance for understanding ecosystem processes, (iii) to small bacteria, eukaryotic phytoplankton, and viruses, i.e., microbes lacking distinct morphologies, and finally, (iv) to a more consistent taxonomic identification. Efficient monitoring systems are characterized by continuous sampling and rapid handling, processing, and analyses of samples. Today, available HTS techniques enable simultaneous analyses of thousands of microbial samples with sufficient sequencing depths to reliably capture taxonomic diversity (Caporaso et al. 2011).

**Fig. 1:**
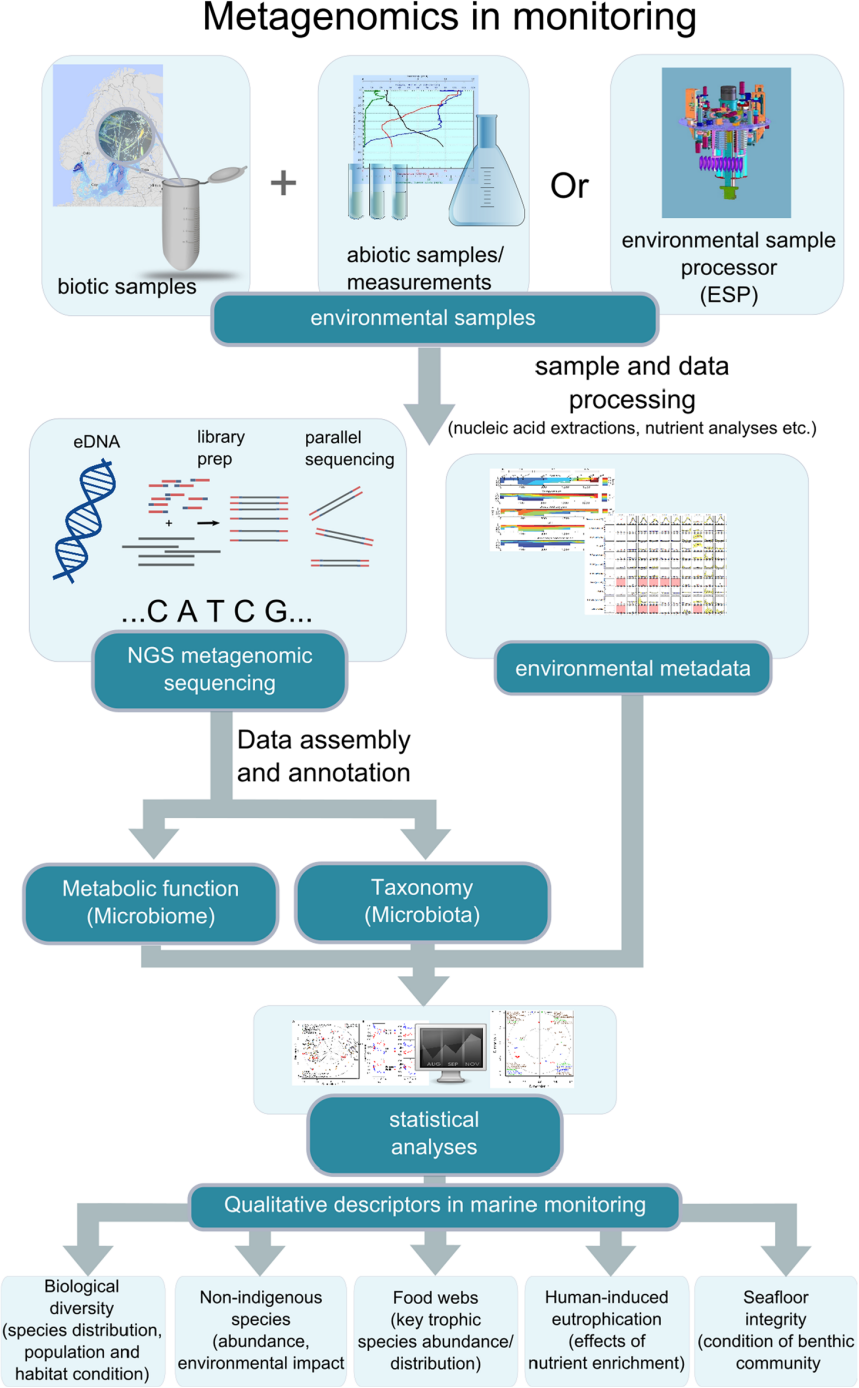
Metagenomics in identifying and monitoring of microbes in the Baltic Sea. A schematic flowchart of the metagenomic approach used in the MiMeBS program and its potential integration in monitoring programs for the Baltic Sea. Criteria for which genomic methods can be used to assess environmental status were derived from the “Marine Strategy Framework Directive” (Bourlat et al. 2013)

Initiatives to introduce genomic/metagenomic analyses in global monitoring programs and observatories are well underway, although not yet a standard (Bourlat et al. 2013; Davies et al. 2014). In an overview of the use of genomic tools in monitoring programs, Bourlat et al. (2013) identified thirteen indicators for qualitative descriptors from the “Marine Strategy Framework Directive” (MSFD, 2008/56/EC), for which genomic tools can be implemented (Fig. 1). The descriptor categories include biological diversity, non-indigenous species, food webs, human-induced eutrophication, and seafloor integrity, all of which are of great relevance in assessing the environmental status of the Baltic Sea. More specific examples of potential targets in a Baltic Sea metagenomics-based monitoring program include genes and organisms of importance for eutrophication and nutrient cycles; with processes in focus encompassing, e.g., photosynthesis, nitrification/denitrification, nitrogen fixation, and phosphate uptake/metabolism. Other sets of target organisms/genes may be related to pollution (e.g, biodegradation of organic pollutants), or to public health concern and “early-warning systems,” including toxin producing microorganisms/toxin genes, as well as pathogens (e.g., *Vibrio*) and pathogenicity genes. Yet another example of microbe/gene sets is those involved in vitamin production in view of the ongoing thiamine (B1) deficiency in higher Baltic Sea organisms (see e.g., Balk et al. 2009). It should, however, be pointed out that while attempts have been made in defining sets of genes that can be used as indicators of environmental perturbations (see Yergeau et al. 2007; Bengtsson-Palme et al. 2014), the field still requires intensive research. Understanding of the relevant reporter genes is necessary for efficient use of metagenomics for monitoring purposes.

**Fig. 2:**
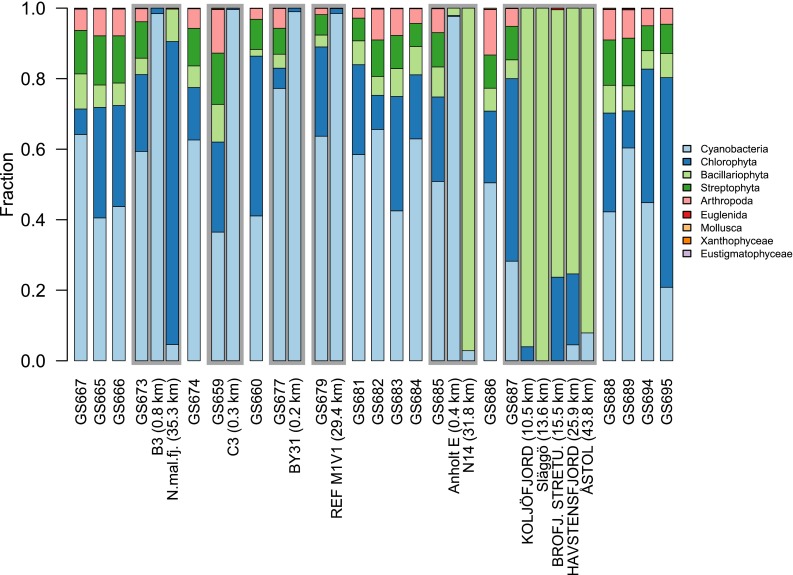
Comparison of phytoplankton and metazoan classifications in environmental samples via genetic (metagenomic) and microscopy-based methods. Samples starting with “GS” represent metagenomic sequencing where classifications were made using similarity searches of protein-coding genes against reference databases. The remaining samples represent microscopy-based classifications available in the SMHI database of environmental parameters (www.smhi.se). SMHI-sites closest to and within 50 km of the “GS” sampling locations were identified and are shown within the same-shaded area as their nearest metagenomic samples with distances shown in *parentheses*. Abbreviated SMHI-sites are as follows: *N.mal.fj* Nordmalingsfjärden, 1, *BY31* BY31 LANDSORTSDJ, *BROFJ. STRETU.* BROFJORDEN/STRETUDDEN

Phytoplankton is since long included in environmental monitoring programs covering the Baltic Sea water body, and methods for sampling and identification were standardized in 1991 through the establishment of the HELCOM phytoplankton expert group. This program, like most others, relies on a morphology-based identification of phytoplankton (light microscopy). However, these practices require considerable work efforts and cover a limited number of samples. Many phytoplankton also lack obvious morphological characteristics needed for identification. The advancement of HTS technologies therefore appears as a promising alternative (or complement) to morphology-based identification. A comparative analysis of a genetic (16S rRNA gene) and a morphology-based identification of phytoplankton revealed considerable discrepancies. For instance, Euglenophyta and Heterokonta were less frequently identified by the sequence-based approach while cyanobacteria were more frequently identified (Eiler et al. 2013). A similar comparison between metagenomic identifications (Dupont et al. 2014) and conventional monitoring data publicly available (SMHI) shows similar discrepancies (Fig. 2). Even though the datasets are not directly comparable (e.g., not based on the same samples), the difference in cyanobacterial, diatom, and green algae identification and abundances are worth noting. These discrepancies are likely indicative of inherent methodological issues for both methods. For the morphology-based identification, these include, for example, cell preservation and human biased microscopic identification, while for the sequence-based method, they relate to DNA/RNA nucleic acid extraction, primer biases, library preparation sequencing depth, and the assembly of short DNA sequences (Gomez-Alvarez et al. 2009; Niu et al. 2010; Schmieder and Edwards 2011). However, the perhaps largest challenge for a sequence-based monitoring program is the limited availability of sequenced reference strains. Metagenomic sequencing projects generate vast amounts of data, but more than half of the reads may end up as “unclassified” due to lack of such reference (genome/genetic) material. One recently introduced way of resolving these issues is the above-discussed assembly or “binning” of genomes using metagenomic sequences (Herlemann et al. 2013; Alneberg et al. 2014; Nielsen et al. 2014). Of specific interest is also genome-sequencing projects focusing on maximizing phylogenetic coverage (Wu et al. 2009; Shih et al. 2013) and accessing the “rare microbial biosphere” (Dini-Andreote et al. 2012). For eukaryotes with larger genomes, DNA barcoding and meta-barcoding are still likely more realistic options for monitoring purposes than both genome sequencing and metagenomics. In the barcoding approach, signature DNA sequences are collected from type-organisms that are either cultured or documented, e.g., by micrographs (Pawlowski et al. 2012). Barcoding initiatives furthermore ensure that sequence-based monitoring data can be harmonized with and constitute a direct continuation of, long-term morphology-based data already at hand.

Novel sequencing systems are currently being developed and evaluated for monitoring purposes in various marine ecosystems, and combined with innovative automated sampling devices such as real-time water monitoring buoys and environmental sample processors (Preston et al. 2009; Ottesen et al. 2011), these are promising tools for both expanding our knowledge and the protection of microbial life. These devices will be particularly important considering ongoing global warming, likely to negatively affect services delivered by natural aquatic ecosystems (Worm et al. 2006; White et al. 2012). Climate change predictions indicate that life in the Baltic Sea will be exposed to more drastic negative effects than those expected in global oceans (Meier et al. 2012), stressing the need for improved sustainable management practices for this body of waters. However, even in a global perspective, research efforts have only recently started to target consequences of such changes for microbial life and hence on the global biogeochemical cycling of nutrients. Expanded genomics-based monitoring programs are therefore as urgent prerequisites for the Baltic Sea as for oceans and other water bodies.

## Conclusion

The ability of an organism to survive through long-term and/or rapid changes in the environment is determined by its genetic repertoire and capacity to adapt physiologically. While metazoans are restricted by long generation times, genetic adaptations in bacteria can be fast and substantial. The steep physicochemical gradients and geographic isolation in the Baltic Sea pose challenging prerequisites for most organisms; however, fast growth and small microbial genomes enable drastic genetic modifications as a response to this variable environment. In addition to potentially profound effects of the microbial biodiversity on productivity and nutrient retention, the large pool of genetic diversity in Baltic Sea microbes discovered through HTS projects provides a valuable resource for resilience. In spite of the documented microbial diversity and functional potential in the Baltic Sea metagenomic datasets generated, many microbial-driven biochemical processes, ecosystem interactions and environmental adaptations remain insufficiently investigated. The studies reviewed here, based on microbial metagenomics, create a necessary base-line for monitoring the effects of a changing environment. Still, in order to develop efficient tools for monitoring programs, additional vertical, horizontal, and seasonal sampling and analyses of microbes in the Baltic Sea are required. Information obtained can then be used to create cost-effective screenings of, e.g., key-players in biogeochemical cycles, pathogenic organisms, and important components of Baltic food webs. This will in turn ensure a scientifically sound, knowledge-based management of the Baltic Sea, and its organismal resources in the future.

## References

[CR1] Alneberg J, Bjarnason BS, de Bruijn I, Schirmer M, Quick J, Ijaz UZ, Lahti L, Loman NJ (2014). Binning metagenomic contigs by coverage and composition. Nature Methods.

[CR2] Andersson AF, Riemann L, Bertilsson S (2010). Pyrosequencing reveals contrasting seasonal dynamics of taxa within Baltic Sea bacterioplankton communities. The ISME Journal.

[CR3] Balk L, Hägerroth PA, Åkerman G, Hanson M, Tjärnlund U, Hansson T, Hallgrimsson GT, Zebuhr Y (2009). Wild birds of declining European species are dying from a thiamine deficiency syndrome. Proceedings of the National Academy of Sciences of the United States of America.

[CR4] Bengtsson-Palme J, Alm Rosenblad M, Molin M, Blomberg A (2014). Metagenomics reveals that detoxification systems are underrepresented in marine bacterial communities. BMC Genomics.

[CR5] Bourlat SJ, Borja A, Gilbert J, Taylor MI, Davies N, Weisberg SB, Griffith JF, Lettieri T (2013). Genomics in marine monitoring: New opportunities for assessing marine health status. Marine Pollution Bulletin.

[CR6] Campbell BJ, Kirchman DL (2013). Bacterial diversity, community structure and potential growth rates along an estuarine salinity gradient. The ISME Journal.

[CR7] Caporaso JG, Lauber CL, Walters WA, Berg-Lyons D, Lozupone CA, Turnbaugh PJ, Fierer N, Knight R (2011). Global patterns of 16S rRNA diversity at a depth of millions of sequences per sample. Proceedings of the National Academy of Sciences of the United States of America.

[CR8] Carstensen J, Conley DJ, Bonsdorff E, Gustafsson BG, Hietanen S, Janas U, Jilbert T, Maximov A (2014). Hypoxia in the Baltic Sea: Biogeochemical cycles, benthic fauna, and management. AMBIO.

[CR9] Davies, N., D. Field, L. Amaral-Zettler, K. Barker, M. Bicak, S. Bourlat, J. Coddington, J. Deck, et al. 2014. Report of the 14(th) genomic standards consortium meeting, Oxford, UK, September 17–21, 2012. *Standards in Genomic Sciences,* 9: 1236–1250.

[CR10] DeLong EF, Preston CM, Mincer T, Rich V, Hallam SJ, Frigaard NU, Martinez A, Sullivan MB (2006). Community genomics among stratified microbial assemblages in the ocean’s interior. Science.

[CR11] Dinasquet J, Kragh T, Schroter ML, Søndergaard M, Riemann L (2013). Functional and compositional succession of bacterioplankton in response to a gradient in bioavailable dissolved organic carbon. Environmental Microbiology.

[CR12] Dini-Andreote F, Andreote FD, Araujo WL, Trevors JT, van Elsas JD (2012). Bacterial genomes: Habitat specificity and uncharted organisms. Microbial Ecology.

[CR13] Dos Santos P, Fang Z, Mason S, Setubal J, Dixon R (2012). Distribution of nitrogen fixation and nitrogenase-like sequences amongst microbial genomes. BMC Genomics.

[CR14] Dupont CL, Larsson J, Yooseph S, Ininbergs K, Goll J, Asplund-Samuelsson J, McCrow JP, Celepli N (2014). Functional tradeoffs underpin salinity-driven divergence in microbial community composition. PLoS One.

[CR15] Eiler A, Drakare S, Bertilsson S, Pernthaler J, Peura S, Rofner C, Simek K, Yang Y (2013). Unveiling distribution patterns of freshwater phytoplankton by a next generation sequencing based approach. PLoS One.

[CR16] Erguder TH, Boon N, Wittebolle L, Marzorati M, Verstraete W (2009). Environmental factors shaping the ecological niches of ammonia-oxidizing archaea. FEMS Microbiology Reviews.

[CR17] Farnelid H, Bentzon-Tilia M, Andersson AF, Bertilsson S, Jost G, Labrenz M, Jürgens K, Riemann L (2013). Active nitrogen-fixing heterotrophic bacteria at and below the chemocline of the central Baltic Sea. The ISME Journal.

[CR18] Feike J, Jürgens K, Hollibaugh JT, Krüger S, Jost G, Labrenz M (2012). Measuring unbiased metatranscriptomics in suboxic waters of the central Baltic Sea using a new in situ fixation system. The ISME Journal.

[CR19] Fernandez C, Farias L, Ulloa O (2011). Nitrogen fixation in denitrified marine waters. PLoS One.

[CR20] Fierer N, Breitbart M, Nulton J, Salamon P, Lozupone C, Jones R, Robeson M, Edwards RA (2007). Metagenomic and small-subunit rRNA analyses reveal the genetic diversity of bacteria, archaea, fungi, and viruses in soil. Applied and Environmental Microbiology.

[CR21] Fonselius S, Valderrama J (2003). One hundred years of hydrographic measurements in the Baltic Sea. Journal of Sea Research.

[CR22] Frias-Lopez J, Shi Y, Tyson GW, Coleman ML, Schuster SC, Chisholm SW, Delong EF (2008). Microbial community gene expression in ocean surface waters. Proceedings of the National Academy of Sciences of the United States of America.

[CR23] Gast V, Gocke K (1988). Vertical distribution of number, biomass and size-class spectrum of bacteria in relation to oxic anoxic conditions in the central Baltic Sea. Marine Ecology Progress Series.

[CR24] Gilbert JA, Field D, Huang Y, Edwards R, Li W, Gilna P, Joint I (2008). Detection of large numbers of novel sequences in the metatranscriptomes of complex marine microbial communities. PLoS One.

[CR25] Giovannoni SJ, Thrash JC, Temperton B (2014). Implications of streamlining theory for microbial ecology. The ISME Journal.

[CR26] Gomez-Alvarez V, Teal TK, Schmidt TM (2009). Systematic artifacts in metagenomes from complex microbial communities. The ISME Journal.

[CR27] Gruber N (2005). Oceanography: A bigger nitrogen fix. Nature.

[CR28] Hagström Å, Larsson U, Hobbie J, Williams PI (1984). Diel and seasonal variation in growth rates of pelagic bacteria. Heterotrophic activity in the sea: NATO conference series.

[CR29] Hajdu S, Höglander H, Larsson U (2007). Phytoplankton vertical distributions and composition in Baltic Sea cyanobacterial blooms. Harmful Algae.

[CR30] Hamersley MR, Turk KA, Leinweber A, Gruber N, Zehr JP, Gunderson T, Capone DG (2011). Nitrogen fixation within the water column associated with two hypoxic basins in the Southern California Bight. Aquatic Microbial Ecology.

[CR31] Heinanen AP (1991). Bacterial numbers, biomass and productivity in the Baltic Sea—A cruise study. Marine Ecology Progress Series.

[CR32] Herlemann DP, Labrenz M, Jürgens K, Bertilsson S, Waniek JJ, Andersson AF (2011). Transitions in bacterial communities along the 2000 km salinity gradient of the Baltic Sea. The ISME Journal.

[CR33] Herlemann DP, Lundin D, Labrenz M, Jürgens K, Zheng Z, Aspeborg H, Andersson AF (2013). Metagenomic de novo assembly of an aquatic representative of the verrucomicrobial class Spartobacteria. AMBIO.

[CR34] Herlemann DP, Manecki M, Meeske C, Pollehne F, Labrenz M, Schulz-Bull D, Dittmar T, Jürgens K (2014). Uncoupling of bacterial and terrigenous dissolved organic matter dynamics in decomposition experiments. PLoS One.

[CR35] Huttenhower C, Gevers D, Knight R, Abubucker S, Badger JH, Chinwalla AT, Creasy HH, Earl AM (2012). Structure, function and diversity of the healthy human microbiome. Nature.

[CR36] Hårdeman F, Sjöling S (2007). Metagenomic approach for the isolation of a novel low-temperature-active lipase from uncultured bacteria of marine sediment. FEMS Microbiology and Ecology.

[CR37] Iverson V, Morris RM, Frazar CD, Berthiaume CT, Morales RL, Armbrust EV (2012). Untangling genomes from metagenomes: Revealing an uncultured class of marine Euryarchaeota. Science.

[CR38] Johannesson K, Andre C (2006). Life on the margin: Genetic isolation and diversity loss in a peripheral marine ecosystem, the Baltic Sea. Molecular Ecology.

[CR39] Johnston AWB, Li Y, Ogilvie L (2005). Metagenomic marine nitrogen fixation—Feast or famine?. Trends in Microbiology.

[CR40] Kahru M, Elmgren R (2014). Multidecadal time series of satellite-detected accumulations of cyanobacteria in the Baltic Sea. Biogeosciences.

[CR41] Karl DM (2007). Microbial oceanography: Paradigms, processes and promise. Nature Reviews Microbiology.

[CR42] Konstantinidis KT, Braff J, Karl DM, DeLong EF (2009). Comparative metagenomic analysis of a microbial community residing at a depth of 4,000 meters at station ALOHA in the North Pacific subtropical gyre. Applied and Environmental Microbiology.

[CR43] Koskinen K, Hultman J, Paulin L, Auvinen P, Kankaanpää H (2011). Spatially differing bacterial communities in water columns of the northern Baltic Sea. FEMS Microbiology and Ecology.

[CR44] Labrenz M, Sintes E, Toetzke F, Zumsteg A, Herndl GJ, Seidler M, Jürgens K (2010). Relevance of a crenarchaeotal subcluster related to *Candidatus* Nitrosopumilus maritimus to ammonia oxidation in the suboxic zone of the central Baltic Sea. The ISME Journal.

[CR45] Larsson J, Celepli N, Ininbergs K, Dupont CL, Yooseph S, Bergman B, Ekman M (2014). Picocyanobacteria containing a novel pigment gene cluster dominate the brackish water Baltic Sea. The ISME Journal.

[CR46] Larsson J, Nylander JA, Bergman B (2011). Genome fluctuations in cyanobacteria reflect evolutionary, developmental and adaptive traits. BMC Evolutionaly Biology.

[CR47] Larsson U, Hajdu S, Walve J, Elmgren R (2001). Baltic Sea nitrogen fixation estimated from the summer increase in upper mixed layer total nitrogen. Limnology and Oceanography.

[CR48] Lauro FM, McDougald D, Thomas T, Williams TJ, Egan S, Rice S, DeMaere MZ, Ting L (2009). The genomic basis of trophic strategy in marine bacteria. Proceedings of the National Academy of Sciences of the United States of America.

[CR49] Lozupone CA, Knight R (2007). Global patterns in bacterial diversity. Proceedings of the National Academy of Sciences of the United States of America.

[CR50] Meier MHE, Andersson HC, Arheimer B, Blenckner T, Chubarenko B, Donnelly C, Eilola K, Gustafsson BG (2012). Comparing reconstructed past variations and future projections of the Baltic Sea ecosystem—First results from multi-model ensemble simulations. Environmental Research Letters.

[CR51] Morris RM, Rappe MS, Connon SA, Vergin KL, Siebold WA, Carlson CA, Giovannoni SJ (2002). SAR11 clade dominates ocean surface bacterioplankton communities. Nature.

[CR52] Nielsen HB, Almeida M, Juncker AS, Rasmussen S, Li J, Sunagawa S, Plichta DR, Gautier L, Pedersen AG (2014). Identification and assembly of genomes and genetic elements in complex metagenomic samples without using reference genomes. Nature Biotechnology.

[CR53] Niu BF, Fu LM, Sun SL, Li WZ (2010). Artificial and natural duplicates in pyrosequencing reads of metagenomic data. BMC Bioinformatics.

[CR54] Ottesen EA, Marin R, Preston CM, Young CR, Ryan JP, Scholin CA, DeLong EF (2011). Metatranscriptomic analysis of autonomously collected and preserved marine bacterioplankton. The ISME Journal.

[CR55] Pawlowski J, Audic S, Adl S, Bass D, Belbahri L (2012). CBOL protist working group: Barcoding eukaryotic richness beyond the animal, plant, and fungal kingdoms. PLoS Biology.

[CR56] Pinhassi J, Hagström Å (2000). Seasonal succession in marine bacterioplankton. Aquatic Microbial Ecology.

[CR57] Pinhassi J, Zweifel UL, Hagström Å (1997). Dominant marine bacterioplankton species found among colony-forming bacteria. Applied and Environmental Microbiology.

[CR58] Preston CM, Marin R, Jensen SD, Feldman J, Birch JM, Massion EI, DeLong EF, Suzuki M (2009). Near real-time, autonomous detection of marine bacterioplankton on a coastal mooring in Monterey Bay, California, using rRNA-targeted DNA probes. Environmental Microbiology.

[CR59] Rheinheimer G, Gocke K, Hoppe HG (1989). Vertical distribution of microbiological and hydrographic-chemical parameters in different areas of the Baltic Sea. Marine Ecology Progress Series.

[CR60] Riemann L, Leitet C, Pommier T, Simu K, Holmfeldt K, Larsson U, Hagström Å (2008). The native bacterioplankton community in the central Baltic Sea is influenced by freshwater bacterial species. Applied and Environmental Microbiology.

[CR61] Savage C, Leavitt PR, Elmgren R (2010). Effects of land use, urbanization, and climate variability on coastal eutrophication in the Baltic Sea. Limnology and Oceanography.

[CR62] Scanlan DJ, Ostrowski M, Mazard S, Dufresne A, Garczarek L, Hess WR, Post AF, Hagemann M (2009). Ecological genomics of marine picocyanobacteria. Microbiology and Molecular Biology Reviews.

[CR63] Schmieder R, Edwards R (2011). Quality control and preprocessing of metagenomic datasets. Bioinformatics.

[CR64] Shih PM, Wu D, Latifi A, Axen SD, Fewer DP, Talla E, Calteau A, Cai F (2013). Improving the coverage of the cyanobacterial phylum using diversity-driven genome sequencing. Proceedings of the National Academy of Sciences of the United States of America.

[CR65] Stal LJ, Albertano P, Bergman B, Bröckel Kv, Gallon JR, Hayes PK, Sivonen K, Walsby AE (2003). BASIC: Baltic Sea cyanobacteria. An investigation of the structure and dynamics of water blooms of cyanobacteria in the Baltic Sea—Responses to a changing environment. Continental Shelf Research.

[CR66] Thureborn P, Lundin D, Plathan J, Poole AM, Sjöberg BM, Sjöling S (2013). A metagenomics transect into the deepest point of the Baltic Sea reveals clear stratification of microbial functional capacities. PLoS One.

[CR67] Väätänen P (1980). Effects of environmental factors on microbial populations in the brackish waters off the southern coast of Finland. Applied and Environmental Microbiology.

[CR68] Venter JC, Remington K, Heidelberg JF, Halpern AL, Rusch D, Eisen JA, Wu DY, Paulsen I (2004). Environmental genome shotgun sequencing of the Sargasso Sea. Science.

[CR69] White C, Halpern BS, Kappel CV (2012). Ecosystem service tradeoff analysis reveals the value of marine spatial planning for multiple ocean uses. Proceedings of the National Academy of Sciences of the United States of America.

[CR70] Worm B, Barbier EB, Beaumont N, Duffy JE, Folke C, Halpern BS, Jackson JBC, Lotze HK (2006). Impacts of biodiversity loss on ocean ecosystem services. Science.

[CR71] Wu D, Hugenholtz P, Mavromatis K, Pukall R, Dalin E, Ivanova NN, Kunin V, Goodwin L (2009). A phylogeny-driven genomic encyclopaedia of Bacteria and Archaea. Nature.

[CR72] Yergeau E, Kang S, He Z, Zhou J, Kowalchuk GA (2007). Functional microarray analysis of nitrogen and carbon cycling genes across an Antarctic latitudinal transect. The ISME Journal.

[CR73] Yooseph S, Sutton G, Rusch DB, Halpern AL, Williamson SJ, Remington K, Eisen JA, Heidelberg KB (2007). The Sorcerer II Global Ocean Sampling expedition: Expanding the universe of protein families. PLoS Biology.

[CR74] Yooseph S, Nealson KH, Rusch DB, McCrow JP, Dupont CL, Kim M, Johnson J, Montgomery R (2010). Genomic and functional adaptation in surface ocean planktonic prokaryotes. Nature.

[CR75] Zinger L, Amaral-Zettler LA, Fuhrman JA, Horner-Devine MC, Huse SM, Welch DBM, Martiny JBH, Sogin M (2011). Global patterns of bacterial beta-diversity in seafloor and seawater ecosystems. PLoS One.

